# The Number of Donor-Specific IL-21 Producing Cells Before and After Transplantation Predicts Kidney Graft Rejection

**DOI:** 10.3389/fimmu.2019.00748

**Published:** 2019-04-09

**Authors:** Nicole M. van Besouw, Lin Yan, Ronella de Kuiper, Mariska Klepper, Derek Reijerkerk, Marjolein Dieterich, Dave L. Roelen, Frans H. J. Claas, Marian C. Clahsen-van Groningen, Dennis A. Hesselink, Carla C. Baan

**Affiliations:** ^1^The Rotterdam Transplant Group, Department of Internal Medicine-Nephrology and Transplantation, Erasmus MC, University Medical Center Rotterdam, Rotterdam, Netherlands; ^2^Laboratory Medicine, West China Hospital, Sichuan University, Chengdu, China; ^3^Department of Immunohematology and Blood Transfusion, Leiden University Medical Centre, Leiden, Netherlands; ^4^The Rotterdam Transplant Group, Department of Pathology, Erasmus MC, University Medical Center Rotterdam, Rotterdam, Netherlands

**Keywords:** IL-21, kidney transplantation, rejection, biomarker, Elispot, end-stage renal disease

## Abstract

Interleukin (IL)-21 supports induction and expansion of CD8^+^ T cells, and can also regulate the differentiation of B cells into antibody-producing plasma cells. We questioned whether the number of circulating donor-specific IL-21 producing cells (pc) can predict kidney transplant rejection, and evaluated this in two different patient cohorts. The first analysis was done on pre-transplantation samples of 35 kidney transplant recipients of whom 15 patients developed an early acute rejection. The second study concerned peripheral blood mononuclear cell (PBMC) samples from 46 patients obtained at 6 months after kidney transplantation of whom 13 developed late rejection. Significantly higher frequencies of donor-specific IL-21 pc were found by Elispot assay in both patients who developed early and late rejection compared to those without rejection. In addition, low frequencies of donor-specific IL-21 pc were associated with higher rejection-free survival. Moreover, low pre-transplant donor-specific IL-21 pc numbers were associated with the absence of anti-HLA antibodies. Donor-reactive IL-21 was mainly produced by CD4^+^ T cells, including CD4^+^ follicular T helper cells. In conclusion, the number of donor-specific IL-21 pc is associated with an increased risk of both early and late rejection, giving it the potential to be a new biomarker in kidney transplantation.

## Introduction

Interleukin (IL)-21 is a pro-inflammatory cytokine and is produced by several T lymphocytes, including CD4^+^, T follicular helper (Tfh) and T-helper 17 (Th17) cells (1, 2). Natural killer T cells (NKT) cells and CD8^+^ T cells can also produce IL-21 (3–5).

IL-21 has a diverse effect on a broad range of immune cells. IL-21 drives T and B cell dependent responses. This pleiotropic cytokine is crucial for T cell-dependent B cell differentiation of antigen-activated naïve and memory B cells into antibody-producing plasma cells (6–8). IL-21 increases cytotoxic activity of NK and NKT cells (9), and the cytokine stimulates Th17 expansion by inducing the expression of the IL-23 receptor (2). IL-21 can stimulate macrophages that induce CD4^+^ T cell proliferation (10). IL-21 also induces proliferation and expression of effector molecules (IFN-γ, granzyme, perforin) in CD8^+^ T cells (11, 12), and facilitates the maturation and maintenance of memory CD8 T cells through STAT3 activation (13). IL-21 can influence the expression of chemokine receptor CX3CR1 and integrin α4β7 on CD8^+^ T cells that results in the accumulation of these aggressive effector and memory CD8 cytotoxic T lymphocytes at non-lymphoid tissues (14). Overproduction of IL-21 occurs in many inflammatory diseases such as rheumatoid arthritis, psoriasis and SLE (15), and is reported to be an important pro-inflammatory cytokine in animal transplant models (16–19).

Studies describing the role of IL-21 after human organ transplantation are promising. The level of serum IL-21 and the IL-21-producing capacity of Tfh cells decrease after kidney transplantation (20, 21), while the number of Tfh cells after transplantation is the highest in renal transplant recipients with pre-existent donor-specific antibodies (DSA) (21). Patients with chronic renal allograft rejection have a characteristic increase in Tfh cells with a decrease in PD1 expression compared to stable patients, while the level of serum IL-21 is comparable between these patient groups (22). In a small cohort of renal allografts, the presence of Th17 cells producing both IL-17 and IL-21 correlated with a shorter graft survival (23). We previously described a positive correlation between the severity of rejection and both IL-21 and IL-21R mRNA expression after heart transplantation (24). IL-21 produced by alloantigen antigen activated cells drives the B cell response (25). We also recently detected IL-21 and Bcl-6 positive cells in renal transplant biopsies obtained during an acute rejection (26).

From the described studies it is clear, that IL-21 plays a role in both cellular and humoral rejection. The Elispot assay enables quantification of donor-reactive IL-21 producing cells (pc) and has proven to be a useful monitoring tool due to its sensitive and accurate detection of rare antigen-specific T-cells and its ability to visualize single positive cells within a population of peripheral blood cells (27–29).

The primary aim of the present study was to investigate whether the number of circulating donor-specific IL-21 pc by Elispot assay before and 6 months after kidney transplantation correlates with the development of both cellular and humoral rejection. In addition, a possible correlation between the number of donor-reactive IL-21 producing cells and the presence of anti-HLA antibodies was determined.

## Materials and Methods

### Patients

All patients participated in an investigator-initiated, prospective, randomized-controlled, parallel group, open label, single center clinical trial that was performed at the Erasmus MC, University Medical Center Rotterdam, the Netherlands. The hypothesis, design and primary outcomes of this trial were published previously (30). The trial was approved by the institutional review board of the Erasmus MC (Medical Ethical Review Board number 2010-080) and the trial was registered in the Dutch national trial registry (http://www.trialregister.nl/trialreg/admin/rctview.asp?TC=2226; number NTR2226, registered February 25, 2010) (30). All patients provided written informed consent in accordance with the declaration of Helsinki. In the present case-control study, PBMCs from 28 patients were available out of 45 patients who developed rejection. PBMCs from 118 patients were available without rejection, 54 patients who remained free from rejection were matched for gender and age with the patients who did develop rejection.

The first group consisted of 35 patients on the waiting list for kidney transplantation. PBMCs were sampled just before transplantation. Fifteen patients developed biopsy-proven acute rejection, as defined by the Banff classification (31), that was treated with anti-rejection therapy. Eleven patients experienced an acute T cell mediated rejection (aTCMR) and four patients had a mixed-type of rejection [aTCMR + active antibody mediated rejection (aABMR)]. The second group consisted of 46 renal transplant recipients who were 6 months after kidney transplantation of which 13 patients developed rejection after 6 months after transplantation. Six of these patients experienced an aTCMR, one patient an aABMR, one patient chronic active ABMR (caABMR), four patients a mixed-type of rejection, and one patient chronic active TCMR (caTCMR). PBMCs were isolated from all 35 patients prior to transplantation and 46 patients at 6 months after transplantation. Patient characteristics are described in Table 1.

**Table 1:** Patient characteristics.

	**Pre-transplantation (*****n*** **=** **35)**			**6 months post-transplantation (*****n*** **=** **46)**		
	**Without rejection (*n* = 20)**	**With rejection (*n* = 15)**	***p*-value**	**Without rejection (*n* = 33)**	**With rejection (*n* = 13)**	***p*-value**
Recipient age (median, range)	57 (37–74)	55 (33–77)	0.73	55 (25–74)	50 (20–68)	0.13
Recipient gender (male) (%)	10 (50%)	11 (73.3%)	0.30	20 (60.6%)	6 (46.0%)	0.51
Caucasian ethnicity (%)	17 (85%)	12 (80%)	1.0	25 (75.5%)	11 (84.6%)	0.70
**CAUSE OF ESRD**						
Diabetes mellitus	3	4		5	2	
Hypertension	4	3		9	2	
Membranous	2	1		4	1	
Nephropathy	1	1		2	1	
FSGS	1	1		0	0	
Interstitial nephritis	5	3		2	0	
Polycystic kidney disease	4	2		11	7	
other						
Living donor (%)	20 (100%)	12 (80%)	0.07	33 (100%)	13 (100%)	1.0
First transplantation (%)	20 (100%)	11 (73.3%)	0.03	31 (93.9%)	12 (92.3%)	1.0
**Total number of HLA mm**	3.25 ± 1.45	4.20 ± 1.08	0.04	3.42 ± 1.46	3.38 ± 1.33	0.84
HLA-A mm	1.00 ± 0.73	1.27 ± 0.70	0.29	1.12 ± 0.60	0.92 ± 0.76	0.37
HLA-B mm	1.15 ± 0.59	1.60 ± 0.63	0.03	1.18 ± 0.68	1.15 ± 0.69	0.89
HLA-DR mm	1.10 ± 0.64	1.33 ± 0.62	0.29	1.18 ± 0.64	1.31 ± 0.48	0.59
**Anti-HLA antibodies**	*n* = 18	*n* = 13		*n* = 29	*n* = 12	
Present (%)	1 (5.5%)	7 (53.8%)	0.002	3 (10.3%)	2 (16.6%)	0.62
**DSA**						
Present (%)	0 (0%)	3 (23.1%)	0.01	2 (6.9%)	1 (8.3%)	1.0

*ESRD, end stage renal disease; FSGS, focal segmental glomerulosclerosis; mm, mismatches; DSA, donor-specific antibodies*.

All patients received induction therapy with basiliximab (Simulect® Novartis Pharma B.V., Arnhem, the Netherlands) and received maintenance therapy consisting of tacrolimus (Prograft® Astellas Pharma, Leiden, the Netherlands), mycophenolate mofetil (MMF: Cellcept® Roche Pharmaceuticals, Woerden, the Netherlands) and prednisolone (30). Prednisolone was tapered from 5 mg daily at month 3 after transplantation to 0 mg in 1 month's time.

### Anti-HLA Antibodies

The complement-dependent cytotoxicity cross-match was negative before transplantation in all patients.

Serum samples from recipients were screened for the presence of HLA antibodies using the Lifecodes Lifescreen Deluxe (LMX) kit, according to the manufacturer's manual (Immucor Transplant Diagnostics Inc. Stamford, CT, USA). Samples that were considered positive, scores 6 and 8 i.e., 2,135 MFI, for either HLA class I (HLA-A, HLA-B, or HLA-C) or HLA class II (HLA-DR or HLA-DQ) antibodies were further analyzed with a Luminex Single Antigen assay, using LABscreen HLA class I and class II antigen beads (One Lambda, Canoga Park, GA, USA) (32).

Briefly, 4 μl of LABscreen beads and 20 μl of serum were mixed in a test well, protected from light. Serum samples were incubated for 30 min at room temperature on a rotating platform (150 rpm), followed by repeated washings with 260 μl wash buffer. Afterwards, each sample was incubated for 30 min with a goat anti-human PE conjugated antibody (1:100 wash buffer) at room temperature, protected from light, and subsequently washed 5 times with wash buffer. Samples were measured using a Luminex 100 reader (Luminex 100, Luminex Corporation, ‘s-Hertogenbosch, the Netherlands) and the baseline normalized values were used. LABscreen negative control serum (LS-NC, One Lambda) was used as a negative control.

### Peripheral Blood Mononuclear Cells (PBMCs) Sampling

PBMCs were isolated from heparinised blood by density gradient centrifugation using Ficoll-Paque (GE Healthcare, Uppsala, Sweden). The PBMCs were collected from the interphase, washed twice, and frozen in RPMI-1640 with glutamax (Life Technologies/Gibco BRL, Paisley, Scotland, United Kingdom) supplemented with 100 IU/ml penicillin (Lonza, Basel, Switzerland), 100 μg/ml streptomycin (Lonza), 15% heat-inactivated human serum, and 10% dimethyl sulfoxide (Merck KGaA, Darmstadt, Germany), and stored at −140°C until use.

### IL-21 Elispot

Polyvinylidene fluoride (PVDF) plates (Millipore, Darmstadt, Germany) were pre-wetted with 70% ethanol during 1 min. After washing the plate, the wells were coated with 50 μl/well anti-human IL-21 mAb (U-CyTech Biosciences, Utrecht, the Netherlands) overnight at 4°C. After washing the wells with PBS, the wells were post-coated with 200 μl/well blocking buffer according to the manufacturer's protocol.

In brief, triplicates of 3 × 10^5^ patient's PBMCs were incubated with 3 × 10^5^ irradiated (40 Gy) PBMCs derived from the donor or 3 × 10^5^ irradiated third-party PBMCs, which were completely HLA-mismatched with donor and recipient, in 200 μl culture medium [RPMI-1640 with glutamax (Life Technologies/Gibco) + 10% heat inactivated FBS (Biowest) + penicillin + streptomycin (100 IU/ml penicillin, 100 IU/ml streptomycin; Lonza)]. Unstimulated patient's PBMC served a negative control. Cells were incubated in the Elispot plate for 44 h at 37°C, 5% CO_2_, and 95% humidity to allow spot formation. Thereafter, the wells were firmly shaked-out and washed with PBS, and 100 μl/well of an appropriately diluted biotinylated anti-human IL-21 detection antibody (U-CyTech Biosciences) was added for a period of 2 h. After washing, the wells were incubated with streptavidin-HRP conjugate (U-CyTech Biosciences) for 1 h followed by AEC substrate (U-CyTech Biosciences) until distinct spots emerged within 30 min. Color development was stopped by washing extensively with water. When the Elispot plates were dry, spots were counted automatically by using a Bioreader 6000 Elispot-reader (BioSys GmbH, Karben, Germany). In case of response in the unstimulated PBMCs, this response was subtracted from the stimulated response.

### Inter- and Intra-assay Variability of IL-21 Elispot Assay

Precision and reproducibility of the donor-specific IL-21 pc frequency was evaluated by calculating the intra-assay and inter-assay variability. In case of intra-assay variability, PBMC samples from 8 transplant patients were tested twice with a 2 weeks interval, and variability was assessed by calculating the coefficient of variation (CV), defined by the ratio of the standard deviation to the mean. CV for Elispot values <10 IL-21 pc/3 × 10^5^ PBMC were not calculated, because of the possibility of too high SD values in the low number of IL-21 pc. The median intra-assay CV was 3.10% (range: 0.00–26.11; Supplementary Table 1). The inter-assay variability was determined in PBMC samples from 12 patients. Two independent operators determined the number of donor-specific IL-21 pc. The median inter-assay CV was 8.00% (range: 1.03–37.94).

### Immunostaining of PBMC by Flow Cytometry

To determine the percentage of different T cell subsets, at least 1 × 10^6^ PBMCs were suspended in isoflow sheath fluid (Beckman Coulter) and stained in a DuraClone IM T cell tube (Beckman Coulter, Miami, FL) according to the manufacturer's protocol. The T cell tube contained anti-CD45, CD3, CD8, CD4, CD45RA, and CCR7. Samples were measured by use of the Navios flow-cytometer (Beckman Coulter).

To determine the Tfh, at least 1 × 10^6^ PBMC were washed by Fascflow (BD Biosciences, New Jersey, US) and stained with CD3 BV510 (Biolegend, California, US), CD4 BV421 (Biolegend, California, US), CXCR5 Alexa Fluor 647 (BD Biosciences, New Jersey, US), and PD1 APC-Cy7 (Biolegend, California, US) for 30 min at room temperature in the dark.

### CD4^+^ and CD8^+^ Cell Isolation

CD4^+^ and CD8^+^ cells were separated from PBMCs by using the CD4^+^ T cell and CD8^+^ T cell isolation kit from Miltenyi Biotec GmbH, Bergisch Gladbach, Germany. The CD4^+^ and CD8^+^ cells were isolated according to the manufacturer's protocol “depletes.” The autoMACS Pro Separator (Miltenyi) was used to collect the two T cell subsets. The purity of both populations was >95%.

### Mixed Lymphocyte Reaction and Immunostaining

Patient's PBMC were stimulated for 3 days at 37°C, 5% CO_2_ and 95% humidity with CSFE-labeled, irradiated (40 Gy) donor PBMCs, depleted for CD3^+^ T cells, in culture medium. At the end of day 2 monensin and brefeldin A (GolgiStop and GolgiPlug, BD Biosciences) were added for 16 h in a concentration of 1:1,500 and 1:1,000, respectively, to allow the measurement of intracellularly accumulated cytokines in PBMCs. Thereafter, intracellular IL-21 was measured, and surface marker staining was used to investigate which subsets produced these cytokines. Monoclonal antibodies used for surface marker staining and intracellular cytokine staining were CD3 BV510 (BioLegend), CD4 BV421 (BioLegend), CD8 PerCP (BD Biosciences), CXCR5 APC (BD Biosciences), PD-1 APC-Cy7 (BioLegend), and IL-21 PE (Biolegend).

### Statistical Analysis

Statistical and graphical analysis were performed using SPSS 21.0 (SPSS Inc, Chicago, IL, US) and GraphPad Prism version 5.01 (GraphPad, Inc., La Jolla, CA). Fisher's exact test was used for comparisons between patients with and those without rejection. The Mann-Whitney *U*-test was used to analyze differences in phenotype and the number of IL-12 pc between rejectors and non-rejectors. The Wilcoxon signed rank test was used to compare the donor and third-party reactive responses. Data is presented as median and interquartile range. Logistic regression was performed to assess the odds ratio (OR) and 95% confidence interval (CI). Receiver operating characteristic (ROC) curve analysis was used to calculate the cut-off value of number of donor-specific IL-21 pc. Thereafter, Kaplan-Meier survival analysis was performed to assess differences in rejection-free survival between the groups above and below this cut-off value. A two-sided *p*-value ≤ 0.05 was considered significant.

## Results

### Patient Characteristics

The characteristics of patients included are shown in Table 1. No differences were found regarding age, gender, ethnicity, cause of end stage renal disease (ESRD), and percentage living donors in both patient groups. In the pre-transplant cohort, patients who developed rejection were more often recipients of a repeat transplant (*p* = 0.03) and had a higher number of HLA-B mismatches (*p* = 0.03). Patients who developed rejection more frequently had anti-HLA antibodies (*p* = 0.002) and DSA (*p* = 0.01). These differences were not found in the 6-months cohort.

### Phenotype of PBMC Samples

No difference was found in the percentage of CD4^+^ and CD8^+^ T cells in PBMC samples between patients with rejection and without rejection in both patient cohorts (Supplementary Table 2). Also, the percentage of CD4^+^ naïve, central memory, effector memory, and effector memory RA^+^ (EMRA) cells were comparable between the patients who did or did not develop rejection (Supplementary Figure 1 and Supplementary Table 2).

Only in the 6-months samples, the percentage of CD8^+^ naïve T cells (CD8^+^CD45RA^+^CCR7^+^) was higher in the patients who developed late rejection compared to the non-rejection group [median and interquartile range: 45.28% (25.05–54.61) vs. 23.76% (12.14–38.18), *p* = 0.02], while the percentage of CD8^+^ EMRA (CD8^+^CD45RA^+^CCR7^−^) was lower in patients with late rejection compared to patients without rejection [17.63% (10.72–42.84) vs. 36.94% (25.28–49.51), *p* = 0.03]. No difference was found by logistic regression testing the two covariates CD8^+^ naïve T cells and EMRA cells: CD8^+^ naïve T cells, OR = 1.03, 95% CI = 0.99–1.08, *p* = 0.16; CD8^+^ EMRA, OR = 0.97, 95% CI = 0.92–1.02, *p* = 0.29.

In addition, the percentage of Tfh cells (CXCR5^+^PD1^+^) within the CD4^+^ T cell population was not significantly different between patients who developed rejection and those who did not [2.17% (1.35–3.20) vs. 2.08% (1.18–3.36), *p* = 0.81].

### Third-Party Reactive IL-21 Producing Cells

In 71 samples (pre-transplantation: *n* = 25, 6 months: *n* = 46) we measured both the number of donor and third-party reactive IL-21 producing cells. The number of third-party reactive IL-21 pc was significantly higher than the number of donor-specific IL-21 pc [median and interquartile range: 35/3 × 10^5^ PBMC (14–74) vs. 23/3 × 10^5^ PBMC (6–58) *p* = 0.0006] (Supplementary Figure 2). This probably reflects the fact that third-party cells are completely HLA mismatched with the patient and donor, in contrast to the partly HLA matched donor (mean ± SD: donor 3.38 ± 1.41 vs. third-party 5.11 ± 0.79; *p* < 0.0001). There was no difference between third-party reactivity and patients with and without rejection (35/3 × 10^5^ PBMC [5–72] vs. 33/3 × 10^5^ PBMC [15–78], *p* = 0.67).

### Circulating Donor-Reactive IL-21 Producing Cells in Pre-transplant Cohort

**Figure 1:**
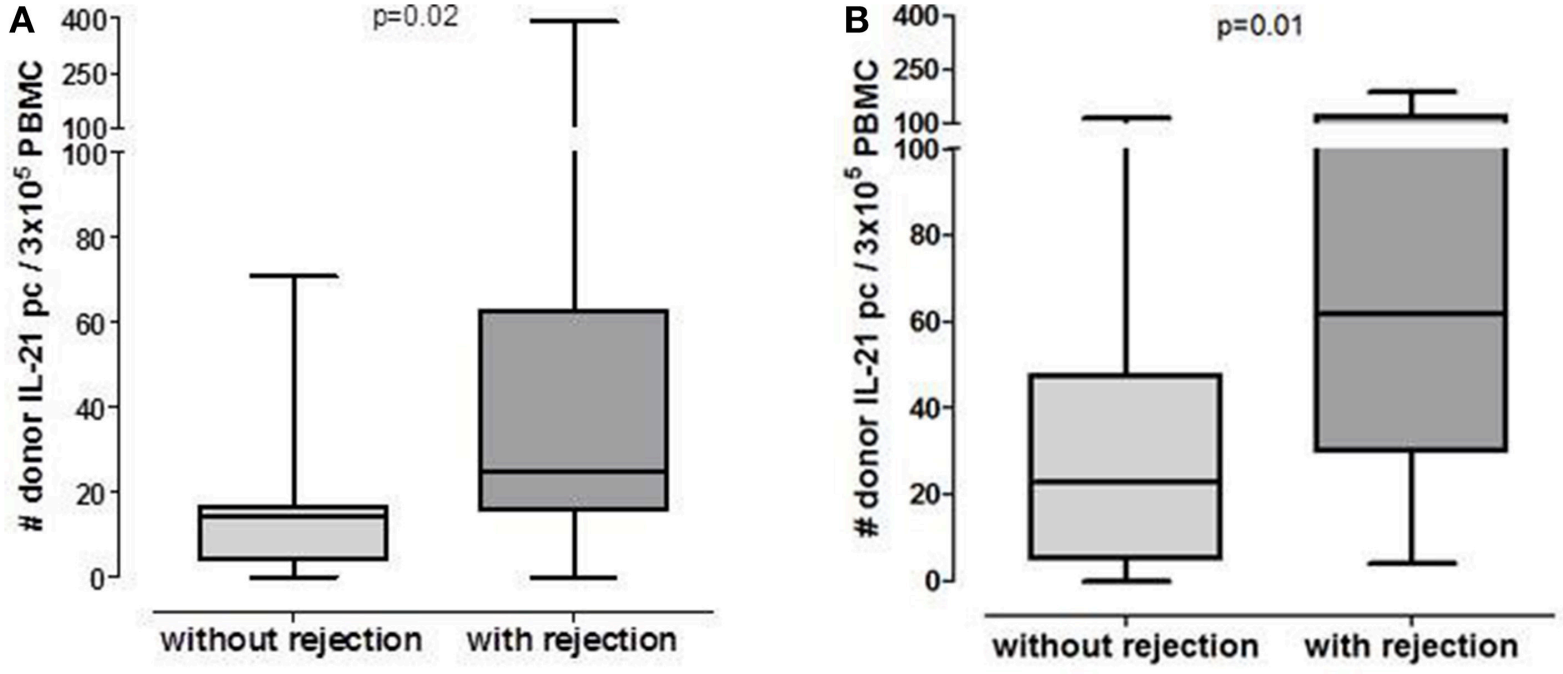
Number of post-transplant donor-specific IL-21 producing PBMC in patients who will or will not develop rejection in pre-transplant cohort (**A**: *n* = 20 without rejection, *n* = 15 with rejection) and 6 months post-transplant cohort (**B**: *n* = 33 without rejection, *n* = 13 with rejection).

Patients who developed an early acute rejection had significantly higher numbers of pre-transplant donor-reactive IL-21 pc compared to patients who did not develop rejection [25/3 × 10^5^ PBMC (16–63) vs. 15/3 × 10^5^ PBMC (4–17), *p* = 0.02, Figure 1A]. Seven patients developed an acute TCMR (aTCMR) grade 1 (*n* = 6 type 1A, *n* = 1 type 1B) (31), and 4 patients an aTCMR grade 2 or 3 (*n* = 2 type 2A, *n* = 1 type 2B, *n* = 3 type 3) (31). Four patients developed a mixed active ABMR (aABMR) and aTCMR (*n* = 1 type 1A, *n* = 2 type 2B, *n* = 1 type 3). No difference was found between type of rejection and the number of donor-reactive IL-21 pc.

**Figure 2:**
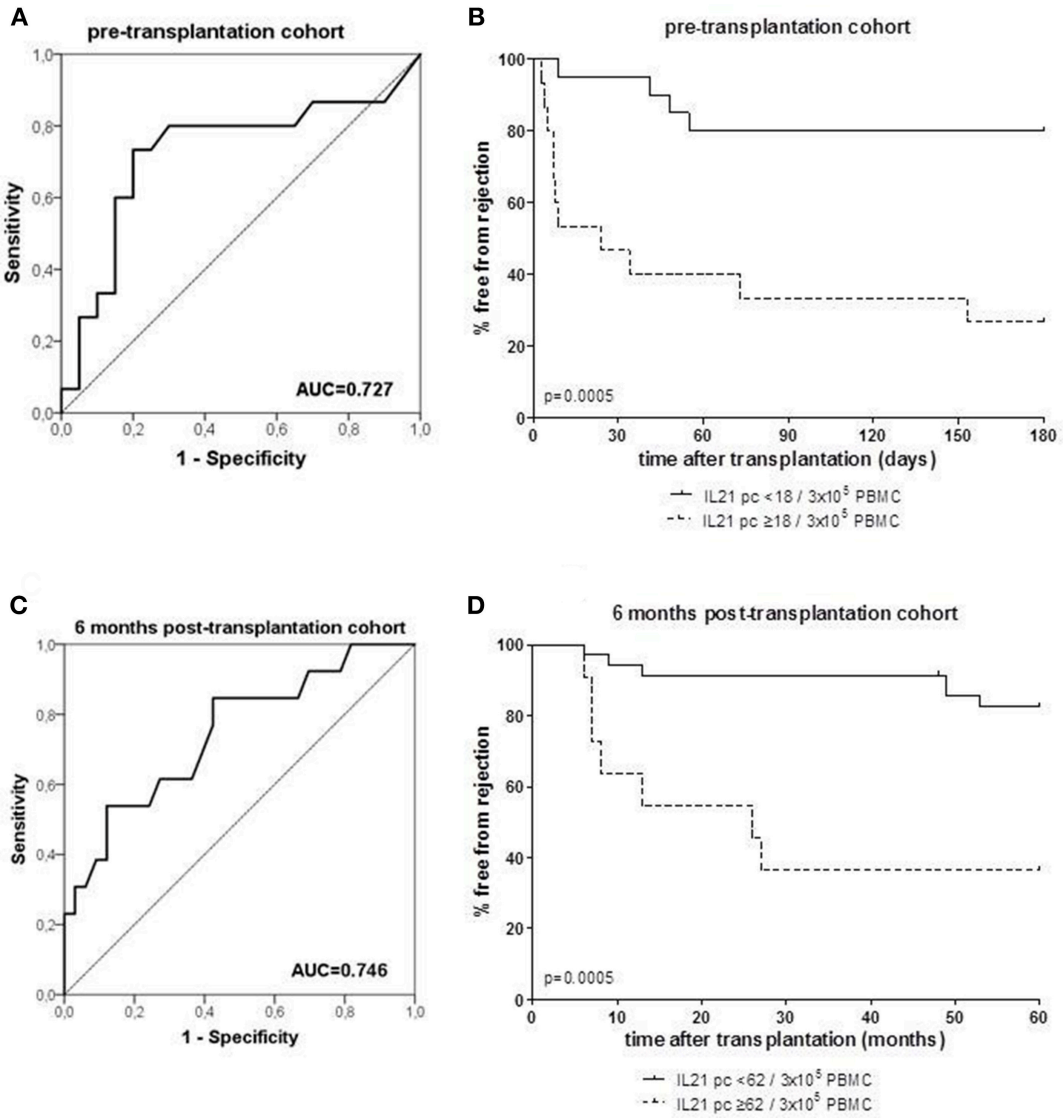
Receiver operating characteristic (ROC) analysis was performed to define the cut-off number of donor-specific IL-21 producing cells (pc) (**A**: PBMC samples taken from patients prior to transplantation; **C**: PBMC samples taken from patient at 6 months after transplantation), and discriminated between patients with and without rejection. A cut-off of 18 spots per 300.000 PBMCs was determined with a specificity of 80% and a sensitivity of 73% in the pre-transplant cohort **(A)**, and a cut-off of 62 spots per 300.000 PBMCs was determined with a specificity of 88% and a sensitivity of 54% in the 6 months post-transplant cohort **(C)**. Thereafter, the percentage of patients with high and low numbers (cut-off values) of IL-21 pc free from rejection were determined in the pre-transplant **(B)** and post-transplant cohort **(D)**. AUC, area under the curve.

ROC analysis showed that at a cut-off value of 18 donor-specific IL-21 pc per 3 × 10^5^ PBMC, discriminated patients with an early rejection from patients without a rejection with a specificity of 80% and a sensitivity of 73% (Figure 2A). Kaplan-Meier survival analysis demonstrates that patients with low numbers of donor-specific IL-21 pc (<18/3 × 10^5^ PBMC) have fewer early rejection episodes compared to those with high numbers (Figure 2B, *p* = 0.0005).

The risk of rejection was associated with the number of transplantations (*p* = 0.03) and the number of HLA mismatches (*p* = 0.04: Table 1). However, an earlier transplant (*p* = 1.0) and the number of HLA mismatches (OR = 1.85, 95% CI = 0.98–3.50, *p* = 0.06) were not associated with the number of donor-specific IL-21 pc. The absence of anti-HLA antibodies prior to transplantation was correlated with the absence of rejection (Table 1, *p* = 0.002), and was associated with low numbers of donor-specific IL-21 pc (OR = 0.05, 95% CI = 0.01–0.50, *p* = 0.01). Although a correlation between DSA and rejection was observed (Table 1, *p* = 0.01), the absence of DSA was not associated with low numbers of donor-specific IL-21 pc (OR = 0.00, *p* = 0.99).

### Circulating Donor-Reactive IL-21 Producing Cells in the 6 Months After Transplantation Cohort

The number of donor-reactive IL-21 pc at 6 months after transplantation was significantly higher in patients who developed rejection compared to those who did not develop rejection [Figure 1B, 52/3 × 10^5^ PBMC (23–106) vs. 23/3 × 10^5^ PBMC (5–48) *p* = 0.01]. Six patients developed aTCMR (*n* = 2 type 1A, *n* = 2 type 1B, *n* = 2 type 3), one patient developed chronic active TCMR (caTCMR), one patient developed aABMR and one chronic, active ABMR (caABMR), and 4 patients developed a combined aABMR and aTCMR (*n* = 1 type 1A, *n* = 2 type 1B, *n* = 1 2A). No association between the number of donor-reactive IL-21 producing cells and the type of rejection was found.

ROC curve analysis suggested that the frequency of donor-reactive IL-21 pc can distinguish rejection from non-rejection with 62/3 × 10^5^ PBMC as a cut-off value with a specificity of 88% and sensitivity of 54% (Figure 2C). Patients with <62 donor-specific IL-21 pc/3 × 10^5^ PBMC had a significantly higher rejection-free survival rate than patients with higher numbers (Figure 2D, *p* = 0.0005).

### IL-21 Producing Cells in CD4^+^, CD8^+^ T Cells, and Tfh Cells

**Figure 3:**
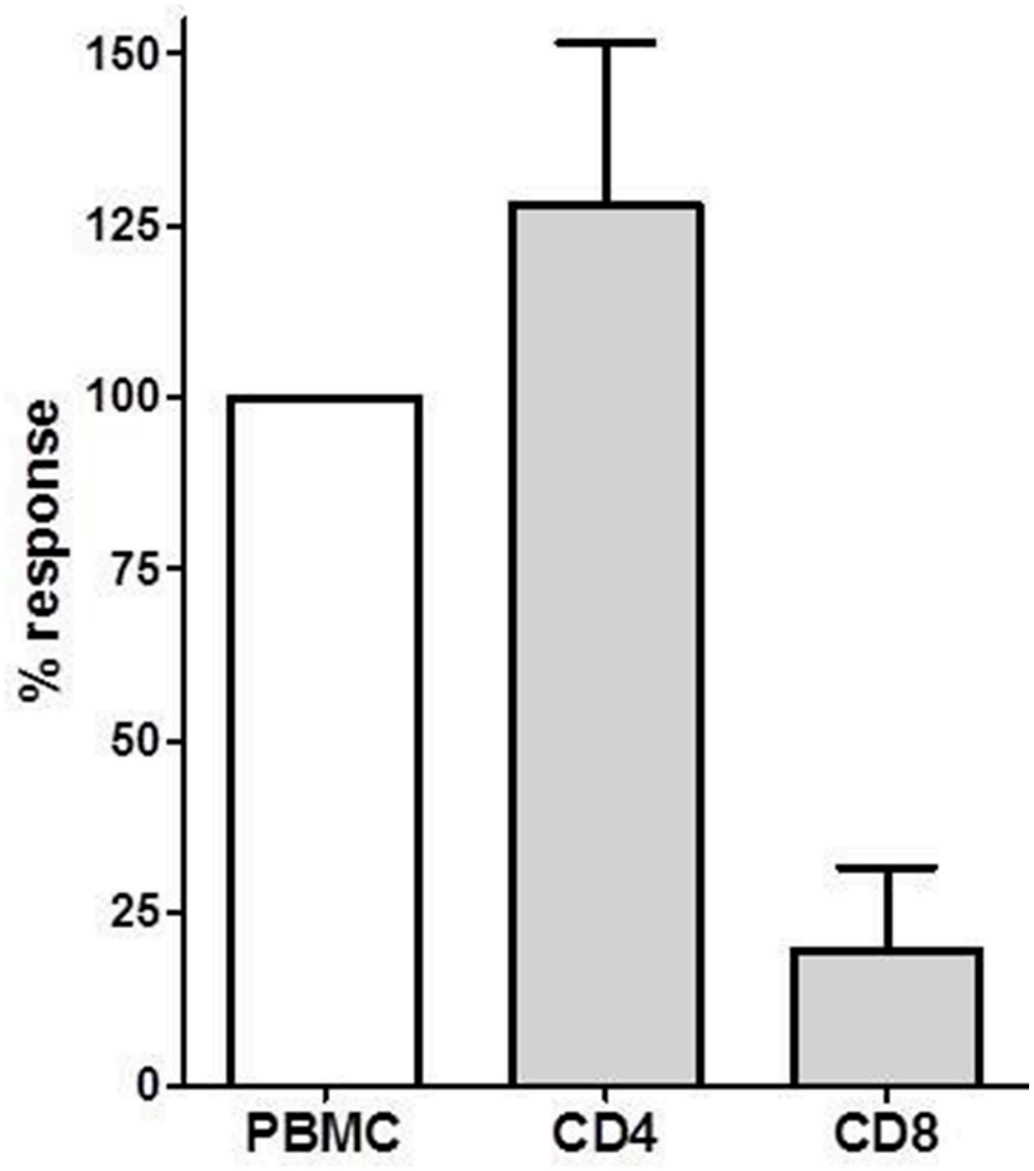
The donor-specific IL-21 producing cell frequency determined in PBMC, CD4^+^ and CD8^+^ T cells in PBMCs of four transplant recipients. The IL-21 response in PBMC was defined as 100%. Mean with SEM is presented.

We isolated CD4^+^ T cells and CD8^+^ T cells to investigate the contribution of these T cell subsets to the IL-21 response. We investigated donor-specific IL-21 reactivity in PBMC, CD4^+^, and CD8^+^ T cells from 4 patients. In all combinations 3 × 10^5^ patient's cells were stimulated with 3 × 10^5^ irradiated donor cells. The response from the PBMC was set as 100%, and compared with the CD4^+^ and CD8^+^ T cell reactivity. Figure 3 depicts that donor-specific IL-21 was mainly derived from the CD4^+^ T cells, while contribution of CD8^+^ T cells to IL-21 production was only minor.

**Figure 4:**
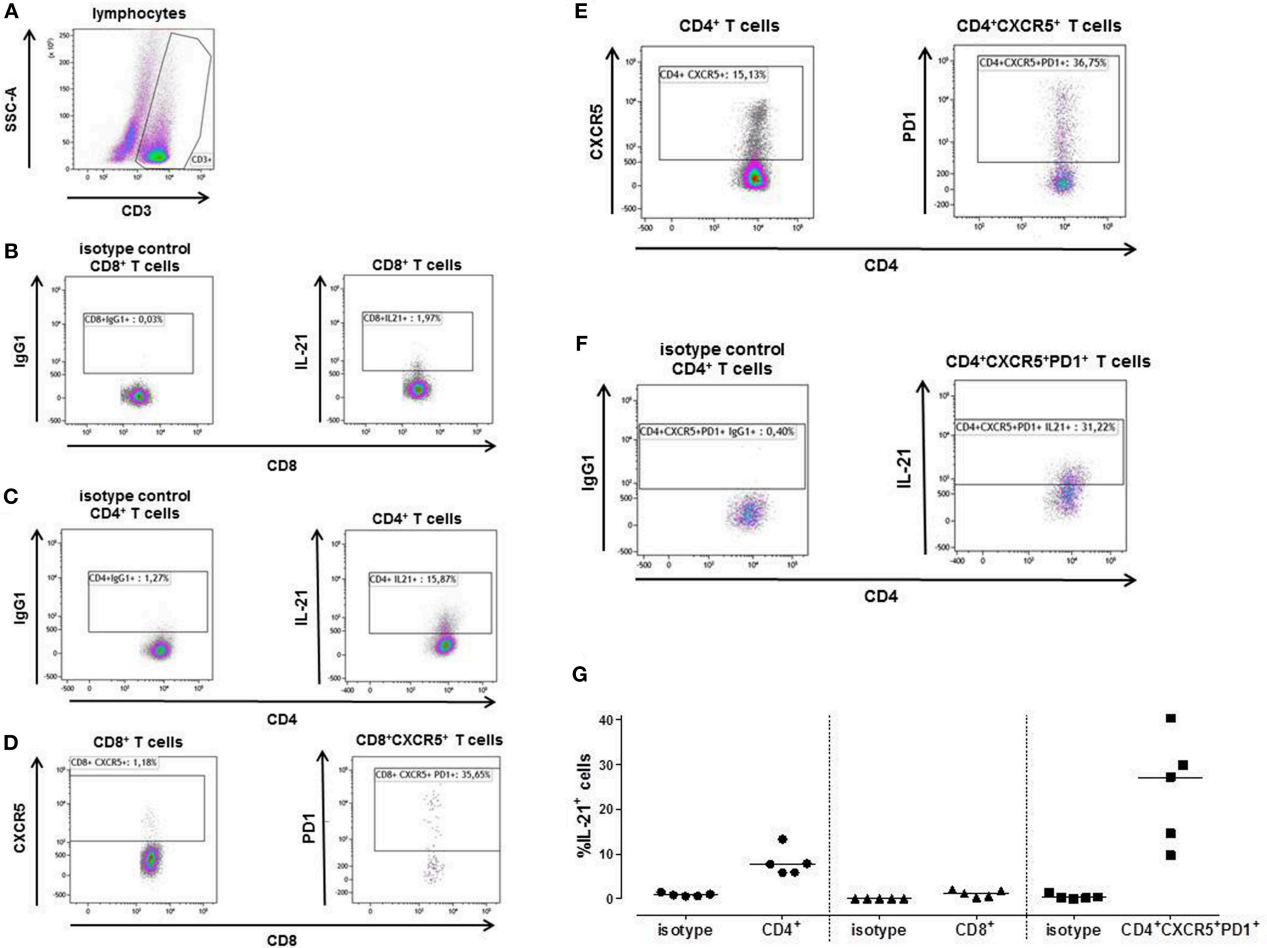
A typical example is depicted for intracellular IL-21 production after stimulation of patient's PBMCs with irradiated donor PBMCs depleted for CD3 **(A)**. The proportion of IL-21 producing cells was determined after 3 days within the CD8^+^ **(B)** and CD4^+^ T cells **(C)**. Tfh cells (CXCR5^+^PD1^+^) were determined within the CD8^+^ **(D)** and CD4^+^ **(E)** T cell population. The percentage of CD8^+^ Tfh cells was too low to be analyzed for IL-21. Within the CD4^+^ Tfh cells 31.22% produced IL-21 **(F)**. The proportion of IL-21 producing cells was determined in PBMC samples from 5 kidney transplant recipients **(G)**. The percentage donor-reactive IL-21 was determined in CD4^+^ and CD8^+^ T cells, and Tfh cells (CD4^+^CXCR5^+^PD1^+^). IgG1 isotypes are presented of each T cell subpopulations.

A mixed lymphocyte reaction (MLR) was performed to determine the percentage IL-21 positive cells amongst the CD4^+^, CD8^+^ and Tfh cells by flow-cytometry. Figure 4 shows a representative example (Figure 4A). Only a few cells from the CD8^+^ T cells (1.97%) were able to produce IL-21 (Figure 4B), while 15.87% of the CD4^+^ T cells did produce IL-21 (Figure 4C). This was comparable with the Elispot results of Figure 3.

1.18% of the CD3^+^CD8^+^ cells expressed CXCR5, from the CD3^+^CD8^+^CXCR5^+^ cells 35.65% also expressed PD1 (Figure 4D). The percentage of CD3^+^CD8^+^CXCR5^+^PD1^+^ cells was too low to be analyzed for IL-21. CD3^+^CD4^+^ cells contained 15.13% CXCR5^+^ cells, 36.75% of these cells were Tfh cells (CD3^+^CD4^+^CXCR5^+^PD1^+^) (Figure 4E). From these CD4^+^ Tfh cells 31.22% contained donor-reactive IL-21 pc (Figure 4F).

We performed this MLR in 5 transplant recipients (Figure 4G). Again, a higher percentage of donor-reactive IL-21 positive cells was found among the CD4^+^ T cells (median, interquartile range; 7.72% [5.88–10.56]) compared to the CD8^+^ T cells (1.16% [0.42–1.90]), and 27.11% (12.16–35.06) of the Tfh cells were able to produce IL-21.

## Discussion

IL-21 is an immunomodulatory cytokine with pleiotropic functions that regulates both the innate and adaptive immune reaction. This cytokine can activate cytotoxic T lymphocytes (CTLs), and can induce immunoglobulin production via stimulation of B cells that transform into plasma producing cells (6–8, 11–14).

The present study demonstrates that the pre-transplant number of donor-specific IL-21 pc could predict early acute rejection after renal transplantation. In addition, the frequency of donor-reactive IL-21 pc 6 months after transplantation predicted late rejection (>6 months after transplantation). Importantly, the number of third-party reactive IL-21 pc did not correlate with rejection. High numbers of donor-reactive IL-21 pc were found in patients who developed both aTCMR and/or aABMR, and no difference was found in the number of donor-specific IL-21 pc between patients with different types of rejection. This suggests that IL-21 is involved in both cellular and humoral allogeneic responses. This could be explained by the broad pleiotropic actions of IL-21. This cytokine modulates the function of both effector cytotoxic CD8 T cells and the development of Th17 cells that can induce cellular rejection (11, 12, 33, 34), and it is also a key factor for the differentiation of B cells into plasma cells that produce DSA resulting in humoral rejection (8). In both the pre-transplant and 6-months post-transplant group, patients with low donor-specific IL-21 pc frequencies had a significantly increased rejection-free survival rate compared to those with high frequencies.

The correlation of donor-specific IL-21 pc and rejection could not be explained by the percentage of Tfh cells (CXCR5^+^PD1^+^ T cells) within the PBMC population, as the percentage of Tfh cells was comparable between patients with and without rejection. In previous studies a correlation with Tfh cells and infection was found (35–37), but each research group uses different phenotypic markers to define these circulating Tfh [e.g., CD4^+^CXCR5^+^ T cells (35), CD4^+^ICOS^+^CXCR3^+^CXCR5^+^ (36), CD4^+^CXCR5^+^CXCR3^+^PD1^low/high^ (37)]. All these cells resemble Tfh cells in peripheral blood. However, different patient groups and different phenotypic markers of these Tfh cells may lead to difficulties in comparing the findings in these studies. This latter has been recognized by others. Schmitt et al. showed a general strategy to define Tfh cells based in cell surface profiles (38). Probably, CXCR5^+^PD1^+^ circulating Tfh cells are not specifically directed to the allograft. Rather, these cells could have a general effect on immune reactivity including anti-viral responses and autoimmune reactions.

The donor-reactive IL-21 response was mainly produced by CD4^+^ cells, because the CD4^+^ T cells contained significantly higher numbers of donor-specific IL-21 pc than the CD8^+^ T cell population both determined by Elispot assay and flow-cytometry. A main producer (27%) of donor-reactive IL-21 are Tfh cells. Therefore, we consider that donor-reactive IL-21 producing cells do not only derive from Tfh cells, but probably activated CD4 T cells and Th17 cells will produce the main part of IL-21 in the Elispot assay (33).

IL-21 can induce the differentiation of CD8^+^ T cells into effector CD8^+^ T cells by increasing granzyme B, perforin, IFN-γ, the chemokine CXCR1, and the integrin α4β7 on effector CD8^+^ cells (11–14). IL-21 has antitumor effects. Recombinant IL-21 given to melanoma and MethA fibrosarcoma mice models delayed tumor progression via increased number of anti-tumor CTLs (39, 40). Remarkably, perforin, and not IFN-γ or other Th1 or Th2 cytokines, is required for the IL-21 antitumor response (39). These data suggest that IL-21 has a unique ability to promote the ability of CD8^+^ T cells to become potent effector CTLs. Therefore, we postulate that IL-21 activates allo-reactive CD8^+^ CTLs that migrate to the allograft resulting in an aTCMR.

The presence of anti-HLA antibodies is a marker for sensitized kidney transplant recipients. These sensitized recipients had higher frequency of donor-specific IL-21 pc. Because interruption of signaling by an IL-21 receptor antagonist abolished the ability to stimulate B cell development and antibody production (25), the high numbers of IL-21 pc present prior to transplantation might be related to the presence of anti-HLA antibodies. High numbers of IL-21 pc might promote anti-HLA antibody formation by providing help to alloantigen activated B cells and an inflammatory response in graft, that results in an aABMR. Unfortunately, not enough aABMR samples were available to confirm this hypothesis. Therefore, further studies are necessary to elucidate the function of IL-21 underlying this process of aTCMR and aABMR.

The importance of IL-21 has already been demonstrated in preclinical studies in autoimmune disease (15, 41, 42). Patients with rheumatoid arthritis, SLE, type 1 diabetes mellitus, and Crohn's disease can participate in phase 1 and 2 clinical trials with anti-IL-21 monoclonal antibody therapy (43). Anti-IL-21 monoclonal antibody therapy could be an promising prophylactic treatment to prevent both aTCMR and aABMR. The early treatment of anti-IL-21 monoclonal antibodies could prevent the invasion of aggressive CTLs into the graft and the development of DSA.

In summary, donor-reactive IL-21 pc determined by Elispot assay could have the potential to predict allograft rejection. The present study has some limitations. Because of the small numbers of rejection samples, differences between aTCMR and aABMR could not be found. A prospective validation study and an external multicentre validation of the number of donor-specific IL-21 pc predicting rejection is advisable. Probably, adding more donor-reactive cytokine producing cells to the study cohort could more clearly discriminate aTCMR from aABMR.

In conclusion, the number of donor-specific IL-21 pc can predict rejection at different phases after kidney transplantation.

## Ethics Statement

All patients participated in a randomized-controlled clinical trial, that was approved by the Medical Ethical Committee of the Erasmus MC (METC 2010-080). This study was conducted in accordance with the declaration of Helsinki. In the present case-control study, we selected 28 patients with rejection from this trial. Patients who did not develop rejection (*n* = 53) were gender and age matched with the patients with rejection.

## Author Contributions

NvB participated in research design, writing of the paper, statistical analysis, performance, and overall supervision of research. LY participated in laboratory experiments, statistical analysis, and writing of the paper. RdK, MK, DR, and MD participated in laboratory experiments. DLR and FC determined HLA typing, HLA antibodies, and reviewed the paper. MC analyzed rejection biopsies and reviewed the paper. DH included the patients, participated in blood sampling, and reviewed the paper. CB participated in research design, interpretation, and writing of the paper.

### Conflict of Interest Statement

The authors declare that the research was conducted in the absence of any commercial or financial relationships that could be construed as a potential conflict of interest.

## Supplementary Material

Supplementary Figure 1 A typical example of the phenotypic analysis of CD4^+^ and CD8^+^ naïve (CD45RA^+^CCR7^+^), central memory (CD45RA^−^CCR7^+^), effector memory (CD45RA^−^CCR7^−^), and EMRA (CD45RA^+^CCR7^−^) T cells in thawed PBMC.

Supplementary Figure 2 Donor and third-party reactive IL-21 producing cells.

Supplementary Table 1 Intra- and inter-assay variability.

Supplementary Table 2 Phenotype of PBMC.
