# Supplementary figures and images for: The Number of Donor-Specific IL-21 Producing Cells Before and After Transplantation Predicts Kidney Graft Rejection
Source: Front Immunol. 2019 Apr 9;10:748. doi: 10.3389/fimmu.2019.00748 (PMC6465545; doi:10.3389/fimmu.2019.00748)

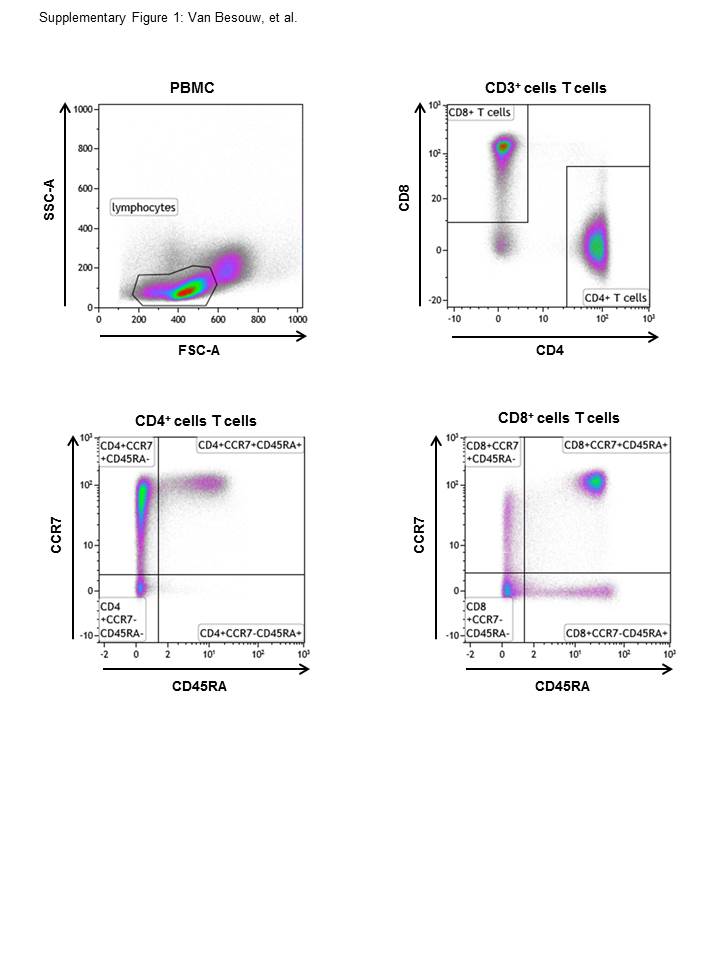

Supplement: Supplementary Figure 1 — A typical example of the phenotypic analysis of CD4+ and CD8+ naïve (CD45RA+CCR7+), central memory (CD45RA−CCR7+), effector memory (CD45RA−CCR7−), and EMRA (CD45RA+CCR7−) T cells in thawed PBMC. [file Image_1.JPEG]

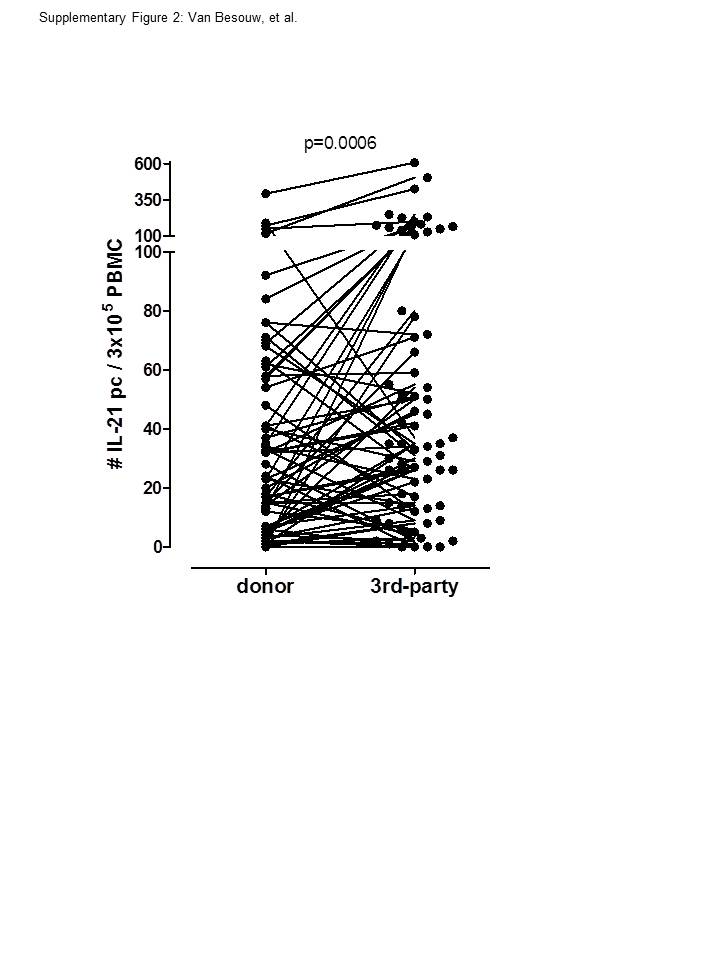

Supplement: Supplementary Figure 2 — Donor and third-party reactive IL-21 producing cells. [file Image_2.JPEG]
