# Supplementary material for: The Number of Donor-Specific IL-21 Producing Cells Before and After Transplantation Predicts Kidney Graft Rejection
Source: Front Immunol. 2019 Apr 9;10:748. doi: 10.3389/fimmu.2019.00748 (PMC6465545; doi:10.3389/fimmu.2019.00748)
Supplement: Supplementary Table 1 — Intra- and inter-assay variability. [file Table_1.DOCX]

**Supplementary Table 1: Intra- and inter-assay variability**

| **Intra-assay variability** | **Measurement 1** | **Measurement 2** | **SD** | **CV in %** |
| --- | --- | --- | --- | --- |
| Patient 1 | 28 | 28 | 0 | 0 |
| Patient 2 | 25 | 25 | 0 | 0 |
| Patient 3 | 53 | 77 | 16.97 | 26.11 |
| Patient 4 | 67 | 70 | 2.12 | 3.10 |
| Patient 5 | 0 | 0 | 0 | nc |
| Patient 6 | 2 | 2 | 0 | nc |
| Patient 7 | 6 | 26 | 14.14 | nc |
| Patient 8 | 19 | 15 | 2.83 | 16.64 |
|  |  |  |  |  |
| **Inter-assay variability** | **Operator 1** | **Operator 2** |  |  |
| Patient 9 | 28 | 25 | 2.12 | 8.00 |
| Patient 10 | 28 | 25 | 2.12 | 8.00 |
| Patient 11 | 53 | 67 | 0.16 | 16.50 |
| Patient 12 | 77 | 70 | 0.07 | 6.73 |
| Patient 13 | 0 | 2 | 1.41 | nc |
| Patient 14 | 0 | 2 | 1.41 | nc |
| Patient 15 | 6 | 19 | 0.74 | nc |
| Patient 16 | 26 | 15 | 0.38 | 37.94 |
| Patient 17 | 162 | 158 | 0.02 | 1.77 |
| Patient 18 | 68 | 69 | 0.01 | 1.03 |
| Patient 19 | 18 | 20 | 0.07 | 7.44 |
| Patient 20 | 54 | 63 | 0.11 | 10.88 |

Two Elispot measurements were performed by the same operator (intra-assay variability) or by two different operators (inter-assay variability). Patient’s PBMC were stimulated with irradiated donor cells. Median number of donor-reactive IL-21 producing cells and standard deviation (SD) from triplicates are shown. Coefficient of variation (CV) for values <10/3x10^5^ PBMC were not calculated (nc).
