# Supplementary material for: The Number of Donor-Specific IL-21 Producing Cells Before and After Transplantation Predicts Kidney Graft Rejection
Source: Front Immunol. 2019 Apr 9;10:748. doi: 10.3389/fimmu.2019.00748 (PMC6465545; doi:10.3389/fimmu.2019.00748)
Supplement: Supplementary Table 2 — Phenotype of PBMC. [file Table_2.DOCX]

**Supplementary Table 2: Phenotype of PBMC**

|  | **pre-transplantation** | | | **6 months post-transplantation** | | |
| --- | --- | --- | --- | --- | --- | --- |
|  | **without rejection** | **with rejection** | **p-value** | **without rejection** | **with rejection** | **p-value** |
| **CD4^+^ T cells** | 59.18%  46.87-65.85 | 59.56%  51.93-70.81 | 0.84 | 58.42%  46.03-67.26 | 65.07%  59.34-71.22 | 0.08 |
| **CD4^+^CD45RA^+^CCR7^+^**  **naïve cells** | 38.96%  22.57-50.86 | 45.89%  30.19-63.44 | 0.54 | 29.35%  20.82-51.46 | 42.11%  34.39-53.47 | 0.12 |
| **CD4^+^CD45RA^-^CCR7^+^**  **central memory cells** | 49.52%  34.96-59.83 | 43.52%  31.06-58.36 | 0.74 | 51.42%  35.07-61.47 | 48.49%  36.80-51.10 | 0.45 |
| **CD4^+^CD45RA^-^CCR7^-^**  **effector memory cells** | 8.14%  4.91-17.55 | 7.62%  2.88-13.87 | 0.46 | 10.47%  6.77-16.21 | 8.20%  5.02-12.67 | 0.29 |
| **CD4^+^CD45RA^+^CCR7^-^**  **EMRA** | 1.02%  0.51-2.41 | 0.99%  0.50-1.35 | 0.54 | 0.79%  0.29-3.85 | 0.49%  0.19-0.92 | 0.09 |
| **CD8^+^ T cells** | 35.22%  29.00-44.78 | 37.59%  26.02-45.35 | 0.93 | 35.97%  27.51-50.22 | 29.43%  25.75-37.37 | 0.09 |
| **CD8^+^CD45RA^+^CCR7^+^**  **naïve cells** | 24.34%  19.52-46.01 | 28.62%  19.98-47.47 | 0.74 | 23.76%  12.15-38.18 | 45.28%  25.06-54.61 | 0.02 |
| **CD8^+^CD45RA^-^CCR7^+^**  **central memory cells** | 17.60%  13.10-22.23 | 11.16%  7.98-22.08 | 0.28 | 10.75%  4.87-17.73 | 9.90%  4.62-16.45 | 0.95 |
| **CD8^+^CD45RA^-^CCR7^-^**  **effector memory cells** | 15.31%  10.03-22.89 | 17.79%  11.78-24.18 | 0.84 | 19.73%  11.59-32.61 | 21.67%  10.86-34.28 | 0.97 |
| **CD8^+^CD45RA^+^CCR7^-^**  **EMRA** | 36.57%  21.60-41.85 | 37.34%  27.38-42.98 | 0.66 | 36.94%  25.28-49.51 | 17.63%  10.72-42.84 | 0.03 |
